# Five years of *European Journal of Psychotraumatology*

**DOI:** 10.3402/ejpt.v7.31350

**Published:** 2016-03-11

**Authors:** Miranda Olff

## Five years ago

Five years ago, the European Society for Traumatic Stress Studies (ESTSS) launched *European Journal of Psychotraumatology* (*EJPT*) (Olff, 2010). Knowing very little about the world of publishing and in particular about Open Access, it was quite an adventure! Somewhat idealistic, and after having been convinced that Open Access is the future publishing model, we performed a detailed search among potential publishers and found Co-Action Publishing, a relatively small Swedish publisher but with a great deal of experience in journals publishing and with a passion for Open Access. We managed to attract highly competent associate editors and to create an editorial board that reflected diversity in culture, background, language, and expertise. And then we started our journal in December 2010.

Over the years, we have been able to attract a rich content covering the whole range of topics related to psychotrauma. The journal has been indexed in major databases (Scopus being the latest); it has been included in PubMed Central and has received a Thomson Reuters Web of Science impact factor (2014: 1.602). It has, in short, become an important stakeholder in the publication domain (see editorials (Olff, 2012, 2013, 2014; Olff & Bindslev, 2011)).

Our readership is increasing and we reach people also in the more remote areas of the world where there is, otherwise, little access to scientific information. Over the years, we have received so many emails of gratitude especially from researchers without academic library access to expensive scientific journals, and also from clinicians. This is certainly what keeps us going.

## Publications over the past 5 years

**Fig. 1:**
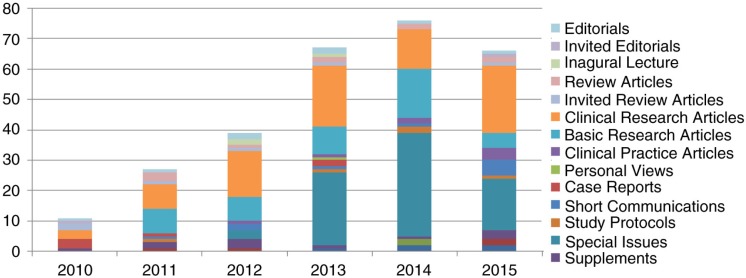
Publications since launch in December 2010.

**Fig. 2:**
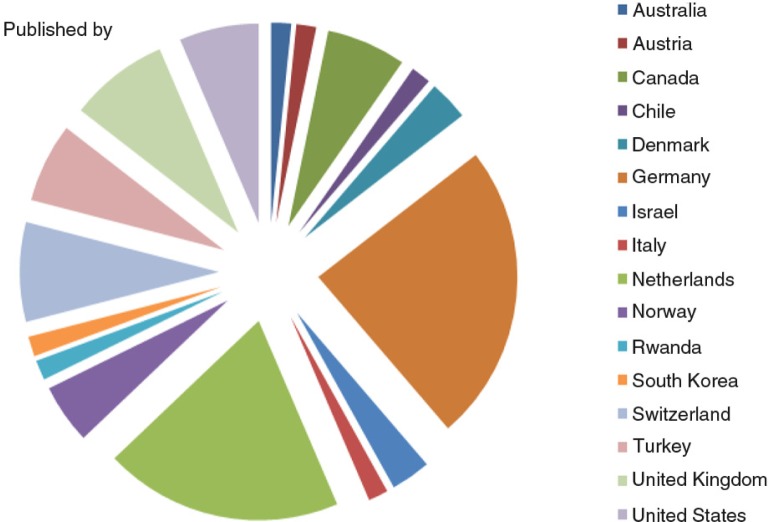
Country of origin of first author of papers published in 2015.

Over the past 5 years, we have seen a steady inflow of papers from a wide range of countries, although the majority is from Europe. Figure 1 shows the number of published papers since the inception of EJPT. Figure 2 shows the geographic distribution of published articles in 2015, showing Germany as the top supplier.

## Special issues

Over the years, we have published a number of special issues. In 2015, there were three: One with contributions from all *EJPT* editors on the gaps in the research—“Trauma and PTSD: Setting the research agenda” (Olff et al., 2015); the second on “Global mental health and trauma” (Purgato & Olff, 2015) with contributions on trauma populations around the world; and the third one covering papers based on keynotes or other main contributions from the ESTSS Conference in Vilnius in June 2015 with one paper already published because it deals with the urgent topic of refugees and we wanted to provide access to it for our readers with no delay (Turner, 2015). In 2015, we also published three supplements; these are proceedings or abstracts from symposia or other scientific meetings: “Psychotrauma update” (Verkes et al., 2015); “Estimating PTSD trajectories” (Van de Schoot, 2015), and “The Swedish Psychotrauma Society scientific conference” (Ranjbar et al., 2015).

## Most accessed articles

Apart from the abstract book of the ESTSS conference in Vienna (2013), the most downloaded articles in 2015 of all articles so far published are shown in Table 1.

**Table 1:** Top 10 most downloaded articles

1. Cloitre et al. (2014)	Distinguishing PTSD, Complex PTSD, and Borderline Personality Disorder: A latent class analysis
2. Taylor (2015)	The influence of shame on posttrauma disorders: Have we failed to see the obvious?
3. Lanius et al. (2015)	Restoring large-scale brain networks in PTSD and related disorders: A proposal for neuroscientifically-informed treatment interventions
4. Rosner et al. (2011)	Treatment of complicated grief
5. Kip et al. (2014)	Accelerated Resolution Therapy for treatment of pain secondary to symptoms of combat-related posttraumatic stress disorder
6. Southwick et al. (2014)	Resilience definitions, theory, and challenges: Interdisciplinary perspectives
7. Dierkhising et al. (2013)	Trauma histories among justice-involved youth: Findings from the National Child Traumatic Stress Network
8. Craparo et al. (2013)	Traumatic experiences in childhood and psychopathy: A study on a sample of violent offenders from Italy
9. Acarturk et al. (2015)	EMDR for Syrian refugees with posttraumatic stress disorder symptoms: Results of a pilot randomized controlled trial
10. Cloitre et al. (2013)	Evidence for proposed ICD-11 PTSD and complex PTSD: A latent profile analysis

It is remarkable to see two 2015 articles in the downloads list. Another recent paper in 2015 that is highly accessed—possibly also due to the full text translations in other languages (to be found in the supplementary materials)—is the article by key representatives of evidence-based interventions for posttraumatic stress disorder (PTSD):

- Schnyder, U., Ehlers, A., Elbert, T., Foa, E., Gersons, B.P.R., Resick, P.A., … Cloitre, M. (2015). Psychotherapies for PTSD: what do they have in common? *European Journal of Psychotraumatology, 6*. doi: http://dx.doi.org/10.3402/ejpt.v6.28186

Most citations according to *Web of Science* JCR in 2014 and 2015 were attracted by Southwick, Bonanno, Masten, Panter-Brick, and Yehuda (2014) and by Cloitre, Garvert, Weiss, Carlson, and Bryant (2014) that were also listed in Table 1 of most downloaded papers. These were closely followed by Elklit, Hyland, and Shevlin (2014) on “Evidence of symptom profiles consistent with posttraumatic stress disorder and complex posttraumatic stress disorder in different trauma samples”; Olff (2015b) on “Mobile mental health: A challenging research agenda”; Armour and Sleath (2014) on “Assessing the co-occurrence of intimate partner violence domains across the life-course: relating typologies to mental health”; Charak et al. (2014) on “Factor structure of PTSD, and relation with gender in trauma survivors from India”; Fodor et al. (2014) on “Is traumatic stress research global? A bibliometric analysis”; and Stenmark, Guzey, Elbert, and Holen (2014) on “Gender and offender status predicting treatment success in refugees and asylum seekers with PTSD.”

Much has been written about refugees in 2015, but there is still very little methodologically sound science that can help us to understand the experiences of some of the most disempowered people. Schock, Rosner, and Knaevelsrud (2015)'s paper, “Impact of asylum interviews on the mental health of traumatized asylum seekers,” examined the effects of legal procedures on people seeking safety and protection in Europe. As well as identifying a rise in intrusive symptoms in the asylum interview group, compared to those not interviewed, they also identified a role of people's perceived justice of the interview, highlighting the interplay of legal and posttraumatic stress factors.

## The business model

Publishing costs money, also Open Access publishing. As we do want to be a golden Open Access journal freely available to any reader around the world, the costs need to be covered by the authors or ideally by their university, research institute, government, or large grant provider as the Welcome Trust or the EU. The EU framework program Horizon 2020, for instance, now requests that research funded with public money is also made freely available to the public, that is, published through an Open Access venue. Recently, in countries like Norway, universities cover the fees of golden Open Access publications immediately. In Europe in October, the European University Association (EUA) agreed on the development of a roadmap to assist European universities in the transition to Open Access.

During the first years of the journal's existence, and with the support of the Netherlands Organisation for Scientific Research (NWO), we were able to run EJPT with no fees for authors. We also later received a grant from the Deutsche Forschungsgemeinschaft (DFG, German Research Foundation), which helped to keep the fees low. Also, since the journal is seen as a membership benefit, ESTSS has been sponsoring the journal since the start. However, we are now slowly transitioning to a journal with a business model that is not dependent on ESTSS or external funders. Compared to other big Open Access publishing houses, the fees are still relatively low, especially for ESTSS members. (By the way, anyone around the world can become an ESTSS member in order to obtain the reduced rates.) We also welcome donations that we will use to waive fees for those who cannot afford to pay. Please visit www.EJPT.net for more information.

## New developments

### Highlights of the article in lay terms

In order to promote the implementation of research and the translation to (clinical) practice, we now introduce “highlights of the article” in the form of a lay summary with bullet points. The message covers the core findings and provides readers with a quick overview of the article. With this we hope to capitalize on the Open Access asset of the journal and reach those in other disciplines who may not be specialized in our type of research but for whom it would nonetheless be relevant; we will also reach those in clinical practice, patients and their families, decision makers, the media, or insurance companies, to name but a few.

### An instruments section

As there is a huge need for information on the right tools or instruments for trauma research or for the best clinical measure to assess patients’ mental health status, we have now introduced a new section on instruments or assessment tools. The aim is to create an authoritative multiple language resource to find the right type of measure for the right type of topic in the right language. Ideally, we would like to have these instruments through Open Access, freely available, without cost or complex copyright issues. Read more about this initiative here (Olff, 2015a).

### Gender policy

From 2016, we will also adopt a gender policy. This means that we follow recommendations of the European Association of Science Editors (EASE) Gender Policy Committee, which works to advance gender- and sex-sensitive reporting and communication in science. Even though we know it is important to discriminate between sex and gender and that single-sex studies may bias our scientific knowledge, still most scientific publications fail to report on potential sex, and gender differences and similarities in studies that include both sexes (EASE).

In EJPT, we will therefore ask authors at least to:

- report the sex of research subjects,
- justify single sex studies,
- discriminate between sex and gender (mostly for human research),
- analyze how sex or gender impact the results, and
- discuss sex and gender issues when relevant

With regard to another mission of EASE which is to “Encourage gender balance among reviewers, on editorial boards, and in editorial offices,” I am pleased to say we are doing quite well.

## Looking ahead

### Special issues

Next year, we will publish a special issue on Eye Movement Desensitization and Reprocessing (EMDR) therapy—a treatment that is increasingly gaining not only popularity but also scientific merits. This issue will critically examine the state of the art with respect to EMDR therapy. We also aim to publish a special issue on the neurobiology underlying development and treatment of psychotrauma related-disorders; several papers are currently under review. This is an area in which we may wish to have stronger growth.

Another quite different but an exciting area is that of Bayesian statistics (Van de Schoot, 2015) and a special issue on this theme is in the making and expected to be published in 2016.

### Where do we want to be in 2020?

At its 10-year anniversary, ESTSS would like to present a journal with articles on the wide range of topics of psychotraumatology, from neurobiology to clinical aspects, which are highly cited and have major clinical and societal impact. We aim to have a financially sound business model with publication fees as the main income but which allows for waivers for those who need it so as not to exclude important contributions from our discourse. We hope to be collaborating with our counterparts worldwide, as we will succeed not by competition but by global collaboration. Psychotraumatology is too important for too many people to not connect and collaborate across all boundaries.

*Miranda Olff* Chief Editor
